# *MTAP* Deletion as a Therapeutic Vulnerability in Cancer: From Molecular Mechanism to Clinical Targeting

**DOI:** 10.3390/ijms262411956

**Published:** 2025-12-11

**Authors:** Paweł Krawczyk, Kamila Wojas-Krawczyk

**Affiliations:** 1 Laboratory of Immunology and Genetics, Chair of Internal Diseases, Medical University of Lublin, 20-059 Lublin, Poland; krapa@poczta.onet.pl; 2 Department of Pneumonology, Oncology and Allergology, Medical University of Lublin, 20-059 Lublin, Poland

**Keywords:** advances malignancies, *MTAP* deletion, MAT2A inhibitors, PRMT5 inhibitors

## Abstract

The *MTAP* (methylthioadenosine phosphorylase) gene, located on chromosome 9p21, plays a crucial role in the methionine salvage pathway and is frequently co-deleted with *CDKN2A* in various malignancies. Loss of *MTAP* expression leads to the accumulation of methylthioadenosine (MTA), which selectively inhibits protein arginine methyltransferase 5 (PRMT5) and creates a unique metabolic vulnerability in *MTAP*-deficient tumors. These alterations have emerged as promising therapeutic targets in precision oncology. Recent advances highlight the potential of exploiting *MTAP* loss through synthetic lethality approaches using PRMT5 and methionine adenosyltransferase 2A (MAT2A) inhibitors. Preclinical and early clinical data indicate that targeting these pathways can selectively impair tumor growth while sparing *MTAP*-proficient cells. Moreover, *MTAP* deletion has been associated with specific molecular and immunologic profiles that may influence treatment response and tumor microenvironment characteristics. This review summarizes current knowledge on the biological functions of *MTAP*, the mechanisms linking its loss to oncogenesis, and the evolving landscape of therapeutic strategies targeting *MTAP*-deficient cancers. Understanding these molecular dependencies offers novel opportunities for the development of precision-based therapies across diverse tumor types.

## 1. Introduction

The search for new therapeutic targets in cancer patients remains a pressing issue. Highly effective molecularly targeted therapies have been developed for patients with non-small-cell lung cancer, melanoma, breast cancer, and others. However, the genetic abnormalities that qualify patients for such therapies are usually rare and/or specific to a single disease (e.g., mutations in the epidermal growth factor receptor gene or rearrangements of the anaplastic lymphoma kinase gene in patients with NSCLC). There are no common molecular factors that would allow for an agnostic treatment selection for patients with different cancers. One such factor is *NTRK* gene rearrangement, which occurs in almost all types of cancer, but its incidence in common cancers (e.g., NSCLC) is very low (less than 1% of patients). Nevertheless, detecting *NTRK* gene rearrangements allows patients, regardless of their cancer type, to qualify for highly effective therapy with larotrectinib or entrectinib. However, there are neoplasms that lack molecular targets for targeted therapies. An example is glial tumors of the central nervous system. Mutations in the *IDH1* and *IDH2* genes or methylation of the *MGMT* gene have been identified in glioblastoma and other types of CNS neoplasms. However, these factors have prognostic value rather than predictive power that qualify for effective treatment (voradisenib has limited efficacy in patients with astrocytoma or oligoastrocytoma with an *IDH* mutation) [1,2,3,4,5,6,7].

One of the most common genetic abnormalities in patients with various types of cancer (including glial tumors of the CNS) is the deletion of the *MTAP* gene. The lack of a functional MTAP enzyme leads to the accumulation of toxic compounds that affect epigenetic phenomena (e.g., histone methylation). However, this effect is not sufficient for cancer cells’ destruction. Enhancing the toxicity caused by MTA in homozygous *MTAP*-deleted cells through molecularly targeted therapies can lead to the effective elimination of cancer cells. This thesis has informed the development of innovative small-molecule drugs, which are being tested in clinical trials in patients with various types of cancer with *MTAP* loss. This offers the opportunity to develop a new, agnostic drug technology for advanced cancer patients [1,2,3,4,5,6,7].

### 1.1. The Molecular Framework of MTAP-Associated Regulation

**Figure 1:**
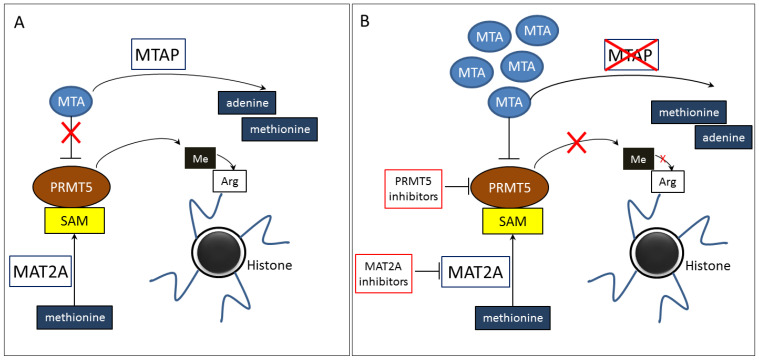
Physiological role of MTAP and PRMT5 enzymes (**A**). Mechanism of action of PRMT5 and MAT2A inhibitors dependent on *MTAP* gene deletion in cancer patients (**B**). MTAP—methylthioadenosine phosphorylase, MTA—methylthioadenosine, PRMT5—protein arginine methyltransferase 5, Arg—argine, Me—methyl residue, SAM—S-adenosylmethionine (PRMT5 cofactor), MAT2A—methionine adenosyltransferase 2A.

The *MTAP* (5-Methylthioadenosine Phosphorylase) gene is located on chromosomal region 9p21.3, near the tumor suppressor gene *CDKN2A* (Cyclin-Dependent Kinase Inhibitor 2A) location. MTAP is a phosphorylase responsible for polyamine metabolism, including that involved in the methionine and adenosine salvage pathways. This enzyme catalyzes the reversible phosphorylation of methylthioadenosine (MTA) to adenine and 5-methylthioribose-1-phosphate. In turn, the *PRMT5* gene is located on chromosome 14 at 14q32.2 locus. PRMT5 (Protein Arginine Methyltransferase 5) is a key enzyme that catalyzes the transfer of a methyl group from S-adenosylmethionine (SAM, AdoMet) to an arginine residue in histone tails or non-histone proteins. SAM is the key methyl donor in cellular processes. The synthesis of SAM from methionine and adenosine triphosphate (ATP) is catalyzed by methionine adenosyltransferase 2A (MAT2A), which transfers adenosyl groups from ATP to L-methionine. Arginine methylation in histones is an epigenetic phenomenon responsible for the chromatin condensation, which affects DNA accessibility and the transcription intensity. Depending on the location of methylated arginine and the degree of methylation in histones tails, different genes expression may be suppressed or increased, that affects the cell proliferation and growth [1,2,3,4,5,6,7] (Figure 1A).

PRMT5 plays a central epigenetic role through the symmetric dimethylation of arginine residues on histones—most prominently H4R3me2s and H3R8me2s—which serves as a critical regulatory mechanism shaping transcriptional programs relevant to oncogenesis. These histone marks are broadly associated with transcriptional repression, and PRMT5-mediated silencing affects the genes involved in differentiation, cell-cycle control, and DNA damage responses. In the context of MTAP deficiency, elevated intracellular MTA acts as a partial competitive inhibitor of PRMT5, leading to a global reduction in symmetric dimethylarginine marks and consequently altering chromatin accessibility and gene expression patterns. This epigenetic rewiring can shift the balance between oncogenic and tumor-suppressive transcriptional networks, sensitizing MTAP-deleted cells to further perturbation of PRMT5 or its upstream regulator MAT2A. Understanding these histone-dependent mechanisms is therefore essential for interpreting the molecular vulnerabilities created by MTAP loss and for rationally deploying PRMT5/MAT2A-targeted therapeutic strategies [1,2,3,4,5,6,7].

### 1.2. Abnormalities in MTAP Gene

Deletion of the *MTAP* gene is one of the most common genetic abnormalities found in neoplasm cells. Moreover, due to the close location of the *MTAP* and *CDKN2A* genes, their co-deletion is often observed in cancers. In patients with pancreatic cancer, *MTAP* deletion often co-occurs with mutations in the *KRAS* gene. However, *MTAP* deletion is less frequent observed in cancer patients with *RB1* mutation. Up to 15% of patients with malignant neoplasms may have a deletion of *MTAP* gene. According to the Center for Cancer Genomics and Advanced Therapeutics (C-CAT) database, *MTAP* deletion is most frequently observed in patients with mesothelioma (33.1%), urothelial carcinoma (23.8%), central nervous system tumors (19.0%), pancreatic cancer (18.4%), cholangiocarcinoma (15.6%), thymomas (15.7%), and non-small-cell lung cancer (14.3%) [8]. However, some studies report as high as 55% incidence of *MTAP* deletion in patients with malignant peripheral nerve sheath tumor, 40% in patients with glioblastoma and pancreatic cancer and 25% in melanoma [9]. This genetic abnormality is rarely observed in patients with prostate cancer (1.2%), colon cancer (1.7%), cervical cancer (3.6%), endometrial cancer (4.0%), breast cancer (4.9%), and seminoma (3.3%) [8]. Mauri et al. confirmed that biliopancreatic and gastro-oesophageal cancers had the highest prevalence of *MTAP* loss (20.5% and 12.7%, respectively); however, in colorectal cancer, *MTAP* deletion was rarely observed (1.1%) [10]. *MTAP* deletion occurs slightly more frequently in men, and the median age of patients at cancer diagnosis is 65 years. In cancer patients, the presence of *MTAP* deletion is a negative prognostic factor associated with shortened overall survival (OS). The data of C-CAT are consistent with other databases such as TCGA (The Cancer Genome Atlas) and GENIE (totaling almost 240,000 cancer patients) [8]. Clouser MC et al. analyzed 352 studies including 37 studies which reported on *MTAP* deletion. Authors showed that *MTAP* deletion prevalence varied across tumor types and were generally the lowest in gastric cancer (4–14%) and the highest in glioblastoma (26–60%) [11]. Some studies suggested a higher risk of death among patients with non-small-cell lung cancer or glioblastoma and with *MTAP* deletions [12].

The deletion of *MTAP* gene leads to the loss of function of the MATP enzyme, which results in the lack of conversion of MTA to adenine. Accumulation of MTA in cancer cells partially blocks PRMT5, causing reduced methylation of histone tails and leading to the modification of chromatin condensation. In this case, the additional blockade of PRMT5 with small-molecule inhibitors appears to be an effective therapeutic approach. Furthermore, the blockade of MAT2A with small-molecule inhibitors leads to reduced SAM synthesis, which also limits arginine methylation in histones. Therefore, the use of MAT2A inhibitors may be an alternative approach to therapy with PRMT5 inhibitors or may enhance the effect of these inhibitors when used in combination therapy. Either way, the effectiveness of PRMT5 and MAT2A inhibitors depends on the accumulation of MTA in cancer cells with *MTAP* gene deletion (Figure 1B) [3,4,5,6,7,9,10,12].

MTAP deletion disrupts one-carbon metabolism by altering methionine and SAM cycling, increases cellular methionine dependency through impaired methylation capacity, and perturbs redox homeostasis by affecting NADPH-generating pathways—all of which collectively reshape the metabolic landscape of MTAP-deficient cells.

Potential metabolic adaptations that bypass the dependence on the MAT2A/PRMT5 axis include alternative methylation sources and downstream effectors that could restore the function of cells with *MTAP* gene deletion. *MTAP* loss can be compensated for by other methyltransferases and by increasing the availability of alternative donors or methyl products. Thanks to this, cells with *MTAP* deletion can activate alternative methylation pathways that bypass MAT2A/PRMT5 axis. In cancer cells, an increase in MAT2A expression is observed, which weakens the inhibitory effect of MTA on PRMT5 and slightly increases the histone methylation process in cells with *MTAP* deletion. Understanding the adaption of processes to the increased MTA concentration in cancer cells with *MTAP* deletion may be crucial for understanding the causes of resistance to PRMT5 and MAT2A inhibitor therapies [10,12,13].

Growing evidence indicates that MTAP deficiency profoundly reshapes the tumor immune microenvironment, driving a spectrum of immunosuppressive mechanisms. The accumulation of MTA in the tumor microenvironment leads to the suppression of T cells and loss of their functions. Tumor infiltration by lymphocytes will be reduced in *MTAP*-deleted cancers. Cytokines with anti-tumor properties including GM-CSF, IL-1α, IL-1β, IL-12, and IFN-γ were abundantly secreted in tumors without *MTAP* deletion, while anti-inflammatory cytokines such as RANTES, HGF, VEGF, IL-6, IL-8, and IL-9 were more represented in tissue with *MTAP* deletion. Moreover, cells with *MTAP* deletion exhibited a reduced response to IL-12, as well as type I and II interferons. In addition to a shift in cytokine profiles, tumor cells undergo immune escape through upregulating the expression of immune checkpoints such as programmed death-ligand 1 (PD-L1). Moreover, MTA acts on adenosine receptors, which can inhibit cellular responses by modulating the production of cyclic AMP (cAMP). This creates a “cold” tumor microenvironment responsible for the resistance to immune checkpoint inhibitors, such as anti-PD-1 or anti-PD-L1 antibodies [14,15].

## 2. Diagnosis of *MTAP* Gene Deletion

Various molecular and pathomorphological techniques have been used to detect *MTAP* deletion or loss of protein expression in tumor cells. Regardless of the method, *MTAP* deletion is detected in formalin-fixed paraffin-embedded (FFPE) tissue material, although the latest genetic techniques also enable the examination of free, circulating, tumor DNA from peripheral blood. The cheapest and most accessible technique for examination of MTAP protein expression is immunohistochemistry (IHC), which utilizes labeled antibodies against MTAP. Homozygous *MTAP* deletion results in the loss of MTAP protein in tumor cells, which corresponds with a negative IHC staining. However, some deletions can result in reduced MTAP protein expression, which is detected in tumor cells, but the staining strength is lower than in cells without *MTAP* gene deletion. A similar situation may occur in the case of heterozygous deletion of the *MTAP* gene. Therefore, molecular techniques should be used to diagnose the presence of *MTAP* gene deletions [11,12,13,14,15,16,17].

Until recently, the standard procedure for the detection of 9p21 loss has been fluorescence *in situ* hybridization (FISH). This method is relatively expensive and time-consuming. The FISH technique uses molecular probes complementary to the studied locus and labeled with fluorochromes. The absence of a signal from probes complementary to the examined locus, while maintaining a signal from control probes complementary to the centromeric sequences of chromosome 9, indicates the presence of a deletion. *MTAP* deletion was initially detected by FISH method during the examination of *CDKN2A* gene copy number variation (CNV) [18,19]. However, it has been shown that homozygous *CDKN2A* deletion does not always coexist with *MTAP* deletion. Brune et al. indicated that MTAP deficiency detected by IHC was found in only 28.4% of patients with the MTAP-deficient NSCLC analyzed for *CDKN2A* deletion. In the TCGA database, only 72.9% of NSCLC patients with *CDKN2A* homozygous deletion had a concurrent *MTAP* gene loss [12]. On the other hand, Dono et al. showed excellent overall sensitivity and specificity of MTAP IHC test results as a surrogate of *CDKN2A* homozygous deletion presence (92.3% and 97.5%, respectively) in meningiomas and *IDH*-mutant astrocytomas [16]. Similar results confirming that MTAP expression may be a surrogate marker for homozygous 9p21.3 deletion were obtained by Vlajnic et al. in patients with urothelial carcinoma (UC). Furthermore, the authors found that the lack of MTAP expression was observed more frequently in metastatic lesions than in muscle-invasive and non-muscle-invasive UC [17].

Other molecular techniques, such as comparative genomic hybridization (aCGH), microarrays, and polymerase chain reaction (PCR), have limited utility in *MTAP* gene diagnosis. aCGH and microarrays detected small deletions or insertions by labeling the examined DNA sample and reference DNA sample with different fluorescent dyes. aCGH can identify and quantify changes in DNA copy numbers across the entire genome comparing a test sample to a reference sample. In patients with deletions of the *MTAP* and *CDKN2A* genes, these tests reveal a lack of fluorescence from *MTAP* and *CDKN2A* loci. Simple PCR, and its variants used in real-time PCR technique, can detect *MTAP* deletions using primers complementary to selected *MTAP* gene sequences and primers complementary to housekeeping gene sequences (genes that are essential for cell survival and are expressed at a relatively constant level in all cells). The absence of amplification products from primers complementary to the *MTAP* gene with retained amplification of the housekeeping gene sequences may indicate homozygous *MTAP* deletion. MLPA (Multiplex Ligation-Dependent Probe Amplification) is a technique commonly used to confirm the presence of large deletions in cancer diagnostics. MLPA is PCR-based technique and utilizes several dozen molecular probes that hybridize to adjacent DNA sequences and are then annealed and amplified in a single PCR using a universal primer pair. The products are analyzed by capillary electrophoresis to determine the relative copy number of each target sequence [20,21,22,23,24].

Currently, the most commonly used technique for diagnosis of *MTAP* gene deletion is next-generation sequencing (NGS). The two most common technologies are Ion Torrent (a semiconductor technology that uses the measurement of hydrogen ions released during DNA polymerization and pH changes) and Illumina (a technology that uses fluorescently labeled nucleotides). NGS enables the testing of point mutations, insertions and deletions, gene amplification and gene fusions, and even enables sequencing of all exomes (WES) and the whole genome (WGS). Typically, from a dozen to several hundred genes are tested simultaneously (Comprehensive Genomic Profiling, CGP) in NGS platforms. NGS also enables the testing of free, circulating, tumor DNA (ctDNA) from liquid biopsy. NGS panels used in routine clinical practice allow for the sequencing of selected sequences of several to several dozen genes where genetic abnormalities are most often located (targeted sequencing). These commercial panels usually do not include the *MTAP* gene [11,19,25,26]. To test for CNV of the *MTAP* gene, custom genetic panels should be designed, or it is recommended to use the commercially available CGP panels, e.g., such proposed by FoundationOne (400 Summer Street, Boston, MA, USA). Clouser et al. reported that the most commonly used test type for *MTAP* deletion diagnosis was NGS using the FoundationOne platform (7 of 29 studies) [11].

## 3. Molecularly Targeted Therapies for Cancer Patients with *MTAP* Gene Deletion

Cancer cells with a homozygous deletion of the *MTAP* gene do not degrade MTA, which is accumulated in cells and inhibits the activity of the PRMT5 enzyme. The old PRTM5 inhibitors (onametostat, PRT811, PF-06939999) were initially used in patients without the *MTAP* gene deletion. However, high doses of these drugs caused significant toxicity, primarily hematological. These drugs did not achieve therapeutic doses in patients without *MTAP* deletion. However, the accumulation of MTA in patients with the *MTAP* gene deletion allows for a reduction in the dose of PRMT5 inhibitors. In cells with the *MTAP* deletion, PMRT5 inhibitors and MTA exert a synergistic effect, enabling the selective destruction of cancer cells by reducing arginine methylation in histones and in other proteins regulating gene expression. An alternative approach to therapy in patients with *MTAP* deletion is the use of MAT2A inhibitors, the enzyme responsible for the formation of SAM, a cofactor of PRMT5. These inhibitors also significantly reduce the activity of PRMT5, which was previously inhibited by MTA accumulation [7,27,28,29,30,31,32,33].

## 4. PRMT5 Inhibitors in Clinical Trials

Clinical trials with PMRT5 inhibitors in patients without deletion in the *MTAP* gene have shown limited effectiveness of such treatment. Pemrametostat (GSK3326595) monotherapy had limited clinical activity in heavily pretreated patients with myeloid neoplasms (clinical benefit rate in 43% of patients). A phase I METEOR-1 study showed response to therapy with pemrametostat only in one patient with cervical cancer and in three patients with adenoid cystic carcinoma form the group of 54 patients with advanced solid tumors. Durable stable disease was achieved in patients with bladder cancer and other tumors. Treatment related adverse events (TRAE) were common but manageable. Onametostat (JNJ-64619178), in a phase I clinical trial, induced objective response only in 5.6% (5 of 90) of patients with advanced solid tumors. However, 3 of 26 patients (11.5%) with adenoid cystic carcinoma had a partial response and achieved a median progression-free survival (PFS) of 19.1 months. Moreover, onametostat demonstrated manageable dose-dependent toxicity, mainly thrombocytopenia. PRT811 showed limited efficacy in patients with recurrent high-grade glioma or uveal melanoma (progression in 83% of patients) with an acceptable safety profile. However, the clinical study with the PF-0693999 molecule was terminated for strategic reasons [33,34,35,36].

Due to unsatisfactory study results in patients without favorable predictive factors, a clinical trial of the MTA-cooperative PRMT5 inhibitor in patients with *MTAP* gene deletion has been initiated. Most of these studies are phase I clinical trials aimed at selecting the appropriate drug dose (dose escalation) and are still recruiting. Information on the efficacy of MTA-cooperative PRMT5 inhibitors is provided by preclinical studies [27,28,29,30,31,32,33]. However, four molecules are already undergoing phase II clinical trials in cancer patients with *MTAP* gene deletion. Information on current clinical trials with MTA-cooperative PRMT5 inhibitors is included in Table 1.

Biomarkers that may influence the effectiveness of PRMT5 and MAT2A inhibitors are being intensively studied in all of the clinical trials. The results of these studies are unknown, as most trials are still recruiting patients. It is known that patients with a homozygous deletion of *MTAP* benefit from treatment with these inhibitors. Patients with a heterozygous loss of one allele of the *MTAP* gene would require excessively high doses of PRMT5 and MAT2A inhibitors to achieve a therapeutic effect. The high expression of MAT2A and PRMT5 in cancer cells with a homozygous deletion of the *MTAP* gene appears to be a negative predictive factor of treatment with PRMT5 and MAT2A inhibitors. Similarly, activation of alternative methylation pathways by methylases independent of the MAT2A/PRMT5 axis may be associated with resistance to this group of inhibitors. G2/M cell cycle arrest has been reported to be a positive predictive factor for this therapy. The efficacy of PRMT5 and MAT2A inhibitors would then depend on the expression of proteins that influence the cell cycle, such as cyclin B protein and CDK1. However, it has not been proven that PRMT5 and MAT2A inhibitors are cell-cycle-dependent and act on dividing cells. A study by Jiang et al. found that the combination of PRMT5 and MAT2A inhibitors may induce synthetic lethality by downregulating the PI3K-AKT-mTOR pathway. Active, alternative cell pathways (such as RAS-RAF-MEK-ERK) may reduce the therapeutic effect of these inhibitors. Therefore, the effectiveness of such therapy may depend on mutations in oncogenes such as *KRAS* or *BRAF*, which are among the most common in various types of cancers [13,37].

BMS-986504 (MTRX1719) is the second generation of MTA-cooperative PRMT5 inhibitors designed to preferentially bind to the PRMT5-MTA complex which accumulates in cancer cells with *MTAP* deletion [38]. In contrast, BMS-986504 demonstrated minimal effects in tumor xenografts or hematopoietic cells without *MTAP* deletion. The safety and efficacy of BMS-986504 are being studied in patients with advanced solid tumors, metastatic NSCLC, and advanced glioblastoma with *MTAP* deletions. Early observations indicated numerous objective responses in patients with melanoma, gallbladder adenocarcinoma, mesothelioma, non-small-cell lung cancer, and malignant peripheral nerve sheath tumors in the presence of *MTAP* deletion. These phase I/II studies have shown dose-limiting hematological toxicity. Furthermore, cell culture studies have demonstrated the rationale for the BMS-986504 and immunotherapy combination. BMS-986504 induced activation of cytotoxic T lymphocytes and increased PD-L1 expression on *MTAP* deletion tumor cells [39].

AMG 193 is an MTA-cooperative PRMT5 inhibitor in vitro, AMG 193 preferentially inhibited the MTA-bound PRMT5 enzyme in tumor cells with *MTAP* deletion. AMG 193 inhibited also the growth of multiple tumor xenografts with this deletion. Moreover, AMG 193 showed antitumor activity in combination with chemotherapy or with sotorasib (KRAS inhibitor active towards tumor cells with p.Gly12Cys mutation in *KRAS* gene) *in vitro* and in animal models. The results of the phase I clinical trial with the AMG 193 molecule are now available. A total of 80 patients with advanced solid tumors received AMG 193 in eight dose-exploration cohorts (doses from 40 to 1600 mg of AMG 193). Patients received a median of two prior lines of therapy, and 23 patients received four or more prior lines of therapy. Nine patients with partial response were observed at high doses of AMG 193 (600–1200 mg) across eight tumor types (NSCLC, pancreatic cancer, cholangiocarcinoma, gallbladder cancer, esophageal cancer, melanoma, renal cell carcinoma, and ovarian Sertolie–Leydig cell tumor). In patients treated with these doses of AMG 193, the overall response rate (ORR) was 21.4%. Disease control rate was 54.8%. Median duration of response (DoR) was 8.3 months. Also, ctDNA clearance and complete intratumoral PRMT5 inhibition were observed in some patients. Any grade TRAEs were reported in 68 patients (85.0%), and grade ≥ 3 in 11 patients (13.8%). The most common TRAEs were nausea (48.8%), fatigue (31.3%), and vomiting (30.0%). Hematological toxicities included anemia (15.0%), lymphopenia (6.3%), neutropenia (3.8%), and leukopenia (3.8%). Thrombocytopenia was reported in one patient. Due to a favorable toxicity profile without significant myelosuppression and encouraging antitumor activity across a variety of solid tumors with *MTAP* deletion, phase II clinical trials with AMG 193 have been initiated for patients with advanced solid tumors, advanced or metastatic NSCLC, gastrointestinal cancers, and lymphoma [40,41].

TNG908 selectively binds to the PRMT5-MTA complex, as well as demonstrates brain penetrance in preclinical models. TNG908 demonstrated selectivity and efficacy in about 200 cancer cell lines with *MTAP* deletion independent of their histology (cancers of bladder, CNS breast, heat and neck, pancreas, lung, gastric, uterine as well as mesothelioma, sarcoma, melanoma, leukemia and lymphoma). Durable tumor regressions were noted in about 30% of the *MTAP*-deleted xenograft models, including models with bladder cancer, cholangiocarcinoma, NSCLC, glioblastoma, mesothelioma, leukemia and lymphoma. Tumor regression was maintained after the discontinuation of treatment. In xenograft models, single-dose exposure to TNG908 allows for a 60% drug concentration in the cerebrospinal fluid relative to plasma. TNG908 was tested in patients with *MTAP*-deleted tumors, including glioblastoma, in open-label, dose-escalation and expansion phase I/II study. This clinical trial enrolled 110 out of 190 planned patients with solid tumors, including 33 with glioblastoma. Partial response was reached in several tumor types (e.g., 36% in patients with pancreatic cancer and 11% in NSCLC patients. However, PR was not shown in glioblastoma patients. Moreover, the concentration of TNG908 in the cerebrospinal fluid and the penetration of the drug into the CNS were found to be insufficient compared to preclinical models. TNG908 was well-tolerated at active doses below 900 mg twice daily. Dose-limited toxicities included elevated creatine kinase and aspartate aminotransferase, nausea, fatigue and altered mental status were observed [42]. The lack of satisfactory outcomes in glioblastoma patients resulted in the termination of the study. However, two new molecules with a similar mechanism of action are currently being developed. TNG462 and TNG456 are small molecules with the ability to selectively inhibit PRMT5 and to penetrate the CNS. TNG462 is currently undergoing phase I/II clinical trials [43,44].

## 5. MAT2A Inhibitors in Clinical Trials

MAT2A inhibitors were developed later than PRMT5 inhibitors and only two molecules are in Phase II clinical trials concerning cancer patients with *MTAP* deletion [7]. The full list of clinical trials is available in Table 1.

The most advanced molecule in the MAT2A inhibitors group is AG 270, also known as S095033. It is an oral, reversible, highly potent and selective MAT2A inhibitor that has been shown to be active against *MTAP*-deleted cancer cells in cell cultures and animal models. Konteatis et al. demonstrate that AG 270 substantially reduces SAM levels in cancer cells and selectively blocks proliferation of *MTAP*-null cells both in tissue culture and xenograft tumors. Dose-escalation, phase I study in advanced solid tumors or lymphoma with homozygous *MTAP* deletion was realized and is now terminated due to changes in the study strategy (introduction of combination therapies instead of AG 270 monotherapy in subsequent studies). The patients included in the study suffered from cholangiocarcinoma (seven patients), pancreatic cancer (seven patients), mesothelioma (four patients), and non-small-cell lung cancer (four patients). Two partial responses were observed and one PR ongoing 4.4 months was achieved in patients with neuroendocrine lung cancer undergoing AG 270 monotherapy. Five other patients had stable disease for ≥16 weeks (ranged from 2.0 to 9.9 months). Maximal reduction in plasma SAM concentration ranged from 54 to 70%. Common toxicities include reversible thrombocytopenia, anemia, fatigue, rash, increases in liver enzymes and bilirubin. All toxicities were dose-limiting, and the maximum tolerated dose of AG 270 was determined to be 200 mg once a day [45,46].

IDE397 is a small molecule that acts as MAT2A inhibitor. In preclinical studies on cell lines and xenograft models with *MTAP* gene deletion, IDE397 showed a cytotoxic effect on cancer cells and inhibited the growth of tumors. In animal models, IDE397 reduced the SAM concentration and selectively altered symmetric dimethylarginine (SDMA) concentration. In a phase I expansion study, IDE397 showed good dose-dependent tolerability in cancer patients with *MTAP* deletion. The phase II clinical trial targeted patients with *MTAP*-deleted urothelial cancer and NSCLC. *MTAP* deletion was detected with NGS or IHC techniques. The study evaluated 27 heavily pre-treated patients (17 with NSCLC and 10 with urothelial cancer), who had undergone 2–3 prior treatment lines. Complete response was demonstrated in one patient with urothelial cancer, while partial response was shown in 38% of squamous NSCLC, 22% of lung adenocarcinoma and 30% of urothelial cancer patients. Stable disease was confirmed in 59% of patients and progression only in two patients. ctDNA reduction was observed in all subjects with evaluable samples and in 33% of patients robust > 90% ctDNA reduction was showed. A total of 81% of patients had a rapid molecular response observed in the first evaluation. Median time to response was 2.7 months and median duration of treatment was 6.2 months, however median DoR and PFS are still immature. Serious adverse events of IDE397 therapy were not observed, and the most frequently noted toxicities were fatigue, peripheral neuropathy, decreased appetite, constipation, creatinine increase, nausea, and asthenia [47]. A new clinical trial is underway to investigate the safety and efficacy of combination therapy with IDE397 and sacituzumab-govitecan (an anti-TROP2 antibody conjugated to a topoisomerase inhibitor) in patients with urothelial carcinoma. Approximately 30 patients with *MTAP*-delated urothelial carcinoma will be enrolled in this phase I dose-escalation study and 50 patients in the dose-expansion trial [48].

Some preclinical evidence suggests legitimacy for combining therapy with PRMT5 and MAT2A inhibitors which may produce more robust responses in cancer patients. Two dose-escalation clinical trials are investigating the safety of the combination of AG 270 and TNG462, as well as IDE397 and AMG 193. Also, the combination of GH56 (PRMT5 inhibitor) and GH3 (MAT2A inhibitor) showed synergy in both *in vitro* and *in vivo* models. The low dose of GH31 in combination with the low dose of GH56 achieved similar efficacy as the high dose of GH56 and rapid tumor regression. Both molecules, especially GH31, have a high ability to penetrate the brain. Therefore, this combination demonstrated the potential for treatment of glioblastoma and other cancers with brain metastases [49,50,51].

## 6. Summary

In summary, it should be emphasized that the detection of *MTAP* gene deletion, one of the most frequently observed genetic abnormalities in cancer cells, opens new therapeutic opportunities for cancer patients. A unique mechanism, associated with the accumulation of MTA, which is not degraded by MTAP, sensitizes cancer cells to PRMT5 and MAT2A inhibitors. Phase I and II clinical trials with these inhibitors in patients with homozygous *MTAP* gene deletions are ongoing.

However, implementing PRMT5 and MAT2A inhibitor therapy into clinical practice will face many challenges. We have very early results from phase I and II clinical trials. Therefore, determining the optimal dosage of individual drugs is currently a priority, especially since intensive studies on the pharmacokinetics and pharmacodynamics of PRMT5 and MAT2A inhibitors are still ongoing. This is associated with the emergence of new adverse effects of therapy and the need to develop new methods of treating toxicities. Reliable data on the validity of combination therapy involving two PRMT5 and MAT2A inhibitors, or individual inhibitors with immunotherapy or chemotherapy, are not available. The combination of PRMT5 and MAT2A inhibitors with immune checkpoint inhibitors seems particularly encouraging. It is important to remember that homozygous *MTAP* loss is associated with an immunologically “cold” tumor microenvironment. Therefore, it appears that the use of PRMT5 and MAT2A inhibitors may restore the immunogenicity of tumors with *MTAP* deletion. Therefore, the rationale for combining PRMT5 and MAT2A inhibitors with immunotherapy with anti-PD-1 and anti-PD-L1 antibodies is strong. The role of these inhibitors in cancer therapy is also controversial. Currently, these drugs are used in patients with advanced cancers after the exhaustion of therapeutic options (usually in the third or subsequent lines of treatment). Perhaps PRMT5 and MAT2A inhibitors combined with other treatment methods (immunotherapy, chemotherapy, and molecularly targeted therapies) could be considered as first-line therapy for patients with selected cancers. In the future, combination therapies may be used as adjuvant settings after surgery or as consolidation therapy after chemoradiotherapy. However, we do not know in what type of cancer such therapies could be effective. The only factor qualifying for therapy is the presence of a homozygous deletion of the *MTAP* gene, detected by NGS or FISH. Other predictive factors for therapy with PRMT5 and MAT2A inhibitors have not yet been identified. Extensive genomic and proteomic research is required to identify the biomarkers influencing the efficacy of this therapy. These factors may vary across cancer types with *MTAP* gene deletion. Therefore, the development of different molecular tests based on NGS technology will be necessary for specific cancers.

## Figures and Tables

**Table 1:** Clinical trials with PRMT5 and/or MAT2A inhibitors in cancer patients with *MTAP* gene deletions.

No	NCT Number	Study Title	Drug Name and Targets	Disease	Line of Treatment	Study Status and Phase
1	NCT03361358	Pre-Screening Study to Identify *MTAP* Loss in Advanced Solid Tumors or Lymphoma	No	Advanced solid tumor 9 other than CNS malignancy) or lymphoma	–	Completed, Study type: observational
**PRMT5 inhibitors in clinical trials**						
2	NCT05094336	A Study of AMG 193 in Participants with Advanced *MTAP*-null Solid Tumors (MTAPESTRY 101)	AMG 193 (PRMT5 inhibitor) monotherapy or in combination with docetaxel	Locally advanced or metastatic solid tumors	II and subsequent	Recruiting, Phase I/II
3	NCT06593522	A Phase 2 Study of AMG 193 in Participants with *MTAP*-deleted Advanced NSCLC (MTAPESTRY 201)	AMG 193 (PRMT5 inhibitor)	Advanced NSCLC	II and subsequent	Recruiting, Phase II
4	NCT06333951	Alone or in Combination with Other Therapies in Subjects with Advanced Thoracic Tumors with Homozygous *MTAP*-deletion (Master Protocol) (MTAPESTRY 104)	AMG 193 (PRMT5 inhibitor) monotherapy or in combination with carboplatin, paclitaxel and pembrolizumab or carboplatin, pemetrexed and pembrolizumab or pembrolizumab alone or sotorasib	Advanced or metastatic NSCLC	I, II or subsequent (patients with CNS metastases)	Recruiting, Phase I
5	NCT06360354	A Study Evaluating AMG 193 in Combination with Other Therapies in Participants with Advanced Gastrointestinal, Biliary Tract, or Pancreatic Cancers with Homozygous Methylthioadenosine Phosphorylase (*MTAP*)	AMG 193 (PRMT5 inhibitor) in combination with gemcitabine and nab-paclitaxel or with modified FOLFIRINOX	Advanced gastrointestinal, biliary tract and pancreatic cancers	I and subsequent	Recriuting, Phase I
6	NCT05275478	Safety and Tolerability of TNG908 in Patients with *MTAP*-deleted Solid Tumors	TNG908 (ralometostat) (PRMT5 inhibitor)	Advanced or metastatic solid tumors	No standard treatment possible	Active, not recruiting, Phase I/II
7	NCT05732831	Safety and Tolerability of TNG462 in Patients with *MTAP*-deleted Solid Tumors	TNG462 (PRMT5 inhibitor) monotherapy or in combination with pembrolizumab	Locally advanced solid tumor	No standard treatment possible	Recruiting, Phase I
8	NCT05245500	Phase 1 Study of MRTX1719 in Solid Tumors with *MTAP* Deletion	BMS-986504 (MRTX1719) (PRMT5 inhibitor). In phase II in combination with standard of care therapy in selected solid tumor malignancies	Mesothelioma, NSCLC, malignant peripheral nerve sheath tumors, pancreatic adenocarcinoma, advanced solid tumors	II and subsequent	Recruiting, Phase I/II
9	NCT06855771	A Study of BMS-986504 in Participants with Pre-treated Advanced or Metastatic Non-small Cell Lung Cancer (NSCLC) with Homozygous *MTAP* Deletion	BMS-986504 (MRTX1719) (PRMT5 inhibitor)	Advanced or metastatic NSCLC	No standard treatment possible	Recruiting, Phase II
10	NCT06883747	Clinical Trial of BMS-986504 in Recurrent GBM Patients	BMS-986504 (MRTX1719) (PRMT5 inhibitor)	Advanced glioblastoma	No standard treatment possible	Recruiting, Early phase I
11	NCT06672523	A Study to Evaluate the Mass Balance, Metabolism, Elimination, and Drug Levels of [14C]-BMS-986504 (MRTX1719) in Participants with Advanced Solid Tumors With Homozygous Methylthioadenosine Phosphorylase Deletion	BMS-986504 (MRTX1719) (PRMT5 inhibitor)	Advanced or metastatic solid tumors	No standard treatment possible	Recruiting, Phase I
12	NCT06973863	A Study of PEP08 in Patients with *MTAP*-Del Advanced or Metastatic Solid Tumors	PEP08 (PRMT5 inhibitor) monotherapy or in combination with standard of care therapy in selected solid tumor malignancies	Advanced or metastatic solid tumors	No standard treatment possible	Not yet recruiting Phase I
13	NCT06914128	A First-in-human Study to Learn How Safe BAY 3713372 is and How it Works in Participants with *MTAP*-deleted Solid Tumors	BAY 3713372 (PRMT5 inhibitor)	Advanced or metastatic solid tumors	No standard treatment possible	Recruiting Phase I
14	NCT06796699	A Phase Ia/Ib Clinical Study of GH56 Capsules in Subjects with *MTAP*-Deleted Advanced Solid Tumors	GH56 (PRMT5 inhibitor)	Advanced or metastatic solid tumors	No standard treatment possible	Recruiting, Phase I
15	NCT06968572	Phase I Study of HSK41959 in Solid Tumors with *MTAP* Deletion	HSK41959 (PRMT5 inhibitor)	Advanced or metastatic solid tumors	No standard treatment possible	Recruiting, Phase I
**MAT2A inhibitors in clinical trials**						
16	NCT04794699	Study of IDE397 in Participants with Solid Tumors Harboring *MTAP* Deletion	IDE397 (MAT2A inhibitor) in combination with docetaxel or paclitaxel or sacitzumab govitecan	Advanced or metastatic solid tumors	II and subsequent	Recruiting, Phase I
17	NCT06568614	An Investigational Study of BG-89894 Tablets in Participants with Advanced Solid Tumors	BG-89894 (SYH2039) (MAT2A inhibitor)	Advanced or metastatic solid tumors	No standard treatment possible	Recruiting, Phase I
18	NCT03435250	Study of AG-270 in Participants with Advanced Solid Tumors or Lymphoma with MTAP Loss	AG-270 (MAT2A inhibitor) monotherapy or in combination with gemcitabine or nab-paclitaxel and gemcitabine	Advanced Solid Tumors or Lymphoma	II and subsequent	Terminated, Phase I
19	NCT06414460	Study of ISM3412 in Participants with Locally Advanced/Metastatic Solid Tumors	ISM3412 (MAT2A inhibitor)	Locally advanced or metastatic Solid Tumors	No standard treatment possible	Recriuting, Phase I
**Combined use of PRMT5 and MAT2A inhibitors in clinical trials**						
20	NCT06188702	S095035 as a Single Agent and in Combination in Adult Participants with Advanced or Metastatic Solid Tumors with Deletion of *MTAP*	AG 270 (MAT2A inhibitor), TNG462 (PRMT5 inhibitor)	Advanced or metastatic solid tumors	II and subsequent	Recruiting Phase I/II
21	NCT05975073	A Phase 1/2 Study of AMG 193 in Combination with IDE397 in Participants with Advanced Methylthioadenosine Phosphorylase (*MTAP*)-Null Solid Tumors	AMG 193 (PRMT5 inhibitor), IDE397 (MAT2A inhibitor)	Advanced or metastatic solid tumors, advanced NSCLC	II and subsequent (in NSCLC patients), No standard treatment possible (in other patients)	Active, not recruiting Phase I/II

## Data Availability

No new data were created or analyzed in this study. Data sharing is not applicable to this article.
